# An approach to building Field Epidemiology Training Programme (FETP) trainees’ capacities as educators

**DOI:** 10.5365/wpsar.2018.9.1.010

**Published:** 2018-09-30

**Authors:** Matthew M. Griffith, Ariuntuya Ochirpurev, Takuya Yamagishi, Shingo Nishiki, Baigalmaa Jantsansengee, Tamano Matsui, Kazunori Oishi

**Affiliations:** a Japan, Infectious Diseases Surveillance Center, National Institute of Infectious Diseases.; b Mongolia Country Office, World Health Organization.; c Mongolia FETP, Field Epidemiology Training Department, National Center for Communicable Diseases, Ministry of Health, Mongolia.; d Infectious Diseases Surveillance Center, National Institute of Infectious Diseases.

Field Epidemiology Training Programmes (FETPs), which are modelled after the Centers for Disease Control and Prevention’s Epidemic Intelligence Service programme, began in 1980 and have produced graduates in more than 70 countries, including 12 in the Western Pacific Region. (*1*, *2*) These programmes aim to “build sustainable capacity for detecting and responding to public health threats” and “develop expertise so that disease outbreaks can be detected locally and prevented from spreading.” (*3*) FETPs thus include training in applied epidemiology and public health services. FETP trainees and graduates, however, often have additional responsibilities: mentoring newer trainees, supervising in the field, leading short training courses, facilitating meetings, etc. Programmes therefore must provide trainees with the knowledge and skills to fulfil these responsibilities.

One approach to building trainees’ capacities has recently shown promise in two Western Pacific Region FETPs. The approach employs participatory training methods based on adult learning principles (*4*) and a systematic design based on the Experiential Learning Theory. (*5*) In contrast to traditional lectures, participatory methods recognize that trainees bring unique experiences and knowledge to a training event that should be shared for the group’s benefit. The approach aims to empower participants to define problems from their own experiences, fostering connection to the material in meaningful ways and encouraging participants to collaboratively develop practical solutions that fit their situations. The systematic design (based in the cyclical Experiential Learning Theory) guides participants to articulate their experiences and to reflect on those experiences to understand how they might relate to the topic’s abstract concepts. Next, participants generalize those concepts to multiple situations and apply them in simulated or real scenarios relevant to their work, therefore creating a new experience with which to repeat the cycle. Another benefit of this approach is that it engages people with different learning styles and not only those who learn best by lecture. This approach has been used or advocated in fields such as medical education, (*6*) geography, (*7*) general higher education, (*8*) and health behaviour education. (*9*)

In February 2017, FETP Japan convened a training of trainers (ToT) using and teaching this approach with facilitators and trainees (see **Table 1**). At the conclusion of the ToT, participants evaluated positively both their satisfaction with the event and their change in knowledge; the only negative comments were requests for more time. ToT participants then used the skills and knowledge acquired to redesign the FETP Surveillance Evaluation Project into a series of eight 3-hour workshops based on the new approach. Facilitators believe that trainees in these redesigned workshops have reached a greater depth of understanding of surveillance evaluation, programme evaluation and national surveillance and that the resulting projects have produced improved recommendations for strengthening national surveillance. FETP Japan trainees have used this approach to improve workshop design and facilitation for the annual Rapid Response Training of Surveillance Officers in local public health centres across the country.

**Table 1:** Example lesson plan for a three-day training of trainers employed in Japan and Mongolia, 2017, for building the training capacity of Field Epidemiology Training Programme trainees and supervisors

Session/Topic	Principal Objective(s)	Training Method(s)
Introductions	• Review characteristics of effective trainers. • Self-assess facilitation strengths and identify areas for improvement.	• Found objects icebreaker • Group brainstorm
Characteristics of Effective Trainers	• Recognize techniques for effective verbal and nonverbal communication during a training event. • Practise techniques to demonstrate interest and respect.	• Reflection • Self-assessment
Effective Verbal & Non-verbal Communication	• Develop strategies for managing challenging behaviours learners might display during a workshop.	• Large-group brainstorm • Q&A • Demonstration • Small-group skit
Challenging Behaviours	• Introduce principles of the Experiential Learning Theory. • Discuss how the Theory can help create better trainings.	• Paired role play
Experiential Learning Theory	• Identify Kolb’s four learning styles. • Explain the importance of accommodating the full variety of learning styles in training courses.	• Guided imagery • Discussion • Lecture • Individual worksheet
Adult Learning Styles	• Compare advantages and disadvantages of different training methods. • Practise designing and delivering a training method.	• Kinaesthetic activity • Interactive lecture • Small-group skill activity • Large-group worksheet
Training Methods	• Match training methods to learning styles and Learning Theory stages.	• Small-group game • Demonstration • Discussion • Small-group teach-backs*
Training Best Practices	• Identify and describe best practices for planning a workshop.	• Creative demonstration • Discussion
Icebreakers, Energizers, Breaks and Closers	• Employ icebreakers, energizers, breaks, and closing activities to improve participant focus, engagement, comfort, and learning.	• Lecture • Brainstorm
Training Course Evaluation	• Create a useful, feasible, and accurate training course evaluation plan.	• Panel discussion • Large-group discussion • Interactive lecture
Planning an Effective Training Course	• Create a training plan for a relevant public health training course using the 12 steps.	• Small-group game • Small-group research • Small-group project • Gallery walk
Individual Action Plans	• Review self-assessment and develop an action plan for continuing transfer of knowledge and skills into the workplace.	• Paired discussion • Paired worksheet

* Teach-backs are a training method in which training participants teach the content they have learnt back to the trainers to confirm apprehension of the concepts.

In March 2017, FETP Japan led a ToT on this approach in Ulaanbaatar for Mongolian FETP trainees, graduates and supervisors. Pre- and post-test questionnaires showed a 70% increase in knowledge and attitudes with respect to learning theory and training methods. Additional outcomes included trainees’ demonstrated ability to design and facilitate participatory training activities (observed during practice sessions), the systematic redesign of the Mongolian FETP Introductory Course and the systematic development of the Basic Epidemiology and Public Health Surveillance training course for officers in the Mongolian Frontline, who are staff in health, veterinary, inspection, and emergency management sectors working in local surveillance and trained to improve country capacity to detect, respond to and contain public health emergencies more rapidly. (*10*) Evaluation showed participants’ learning needs and performance requirements in both courses were met. Of the 63 rapid risk assessments conducted in Mongolia in 2017–2018 (to date), 43% were led by provincial rapid response teams trained using this approach, representing a 30% increase in the percentage of provincial teams conducting risk assessments compared to previous periods. Facilitators commented that trainees attending the redesigned Introductory Course were better able to concentrate compared to previous cohorts, particularly during theory-heavy sessions, and that trainees who completed the ToT have been more effective in leading and facilitating technical working group meetings across sectors. The most notable example was a series of multisectorial meetings (April–November 2017) facilitated by Mongolian FETP graduates who had attended the ToT that led to significant adjustments in the legal framework to improve intersectoral coordination and communication during public health emergencies.

In both Japan and Mongolia, the positive effect this approach has had on trainees has been demonstrated in post-ToT evaluations and post-training application of knowledge and skills to redesign training courses and facilitate events. We believe that subsequent events have been more effective than similar events using traditional approaches.

Implementing this approach revealed some challenges: first, the approach requires assessment of participant learning needs and subsequent systematic training design; thus, facilitators must review and redesign curricula for each event. Second, participatory methods can be new and uncomfortable for individuals educated in formal or traditional styles, implying that programmes with longer records and institutional memory may be hesitant to change. Third, systematically evaluating short- and long-term effects of this approach beyond pre- and post-test questionnaires was challenging; therefore, programme administrators should develop careful impact evaluations that begin before training. Finally, the approach requires a facilitator who is skilled and comfortable with participatory methods. It is expected that with each iteration of ToT a new group of skilled facilitators will emerge who can employ these methods and theories in multiple settings, thus creating a positive ripple that will be resource-saving in the long-term. To support these facilitators, programmes should periodically evaluate and re-train them.

In summary, FETPs seeking to further build sustainable capacity and expertise for handling public health threats across their countries’ health sectors should consider incorporating this approach—combining participatory methods and the Experiential Learning Theory—into routine FETP training schedules. Periodic follow-up assessments with re-training opportunities and concurrent outcome and impact evaluations will further the understanding of its potential cost savings and the sharing of achievements and lessons learnt with other FETPs.
